# Training and standardization of general practitioners in the use of lung ultrasound for the diagnosis of pediatric pneumonia

**DOI:** 10.1002/ppul.24477

**Published:** 2019-08-20

**Authors:** Farhan Pervaiz, Shakir Hossen, Miguel A. Chavez, Catherine H. Miele, Lawrence H. Moulton, Eric D. McCollum, Arun D. Roy, Nabidul H. Chowdhury, Salahuddin Ahmed, Nazma Begum, Abdul Quaiyum, Mathuram Santosham, Abdullah H. Baqui, William Checkley

**Affiliations:** 1 Division of Pulmonary and Critical Care, School of Medicine, Johns Hopkins University, Baltimore, Maryland; 2 Department of International Health, Bloomberg School of Public Health, Johns Hopkins University, Baltimore, Maryland; 3 Department of Biostatistics, Bloomberg School of Public Health, Johns Hopkins University, Baltimore, Maryland; 4 Eudowood Division of Pediatric Respiratory Sciences, School of Medicine, Johns Hopkins University, Baltimore, Maryland; 5 Johns Hopkins University‐Bangladesh, Dhaka, Bangladesh; 6 Reproductive Health Unit, icddr,b, Dhaka, Bangladesh

**Keywords:** pneumonia, standardization, training, ultrasound

## Abstract

**Background:**

Pneumonia is a leading cause of death in children of low‐resource settings. Barriers to care include an early and accurate diagnosis. Lung ultrasound is a novel tool for the identification of pediatric pneumonia; however, there is currently no standardized approach to train in image acquisition and interpretation of findings in epidemiological studies. We developed a training program for physicians with limited ultrasound experience on how to use ultrasound for the diagnosis of pediatric pneumonia and how to standardize image interpretation using a panel of readers.

**Methods:**

Twenty‐five physicians participating in the training program conducted lung ultrasounds in all children with suspected pneumonia, aged 3 to 35 months, presenting to three subdistrict hospitals in Sylhet, Bangladesh, between June 2015 and September 2017.

**Results:**

A total of 9051 pediatric lung ultrasound assessments were conducted through 27 months of data collection. Study physicians underwent training and all were successfully standardized, achieving 91% agreement and maintained a sensitivity and specificity of 88% and 92%, respectively, when their diagnosis was compared with experts. Overall kappa between two readers was high (0.86, 95% confidence interval [CI], 0.84‐0.87), and remained high when a third expert reader was included (0.80, 95% CI, 0.79‐0.81). Agreement and kappa statistics were similarly high when stratified by age, sex, presence of danger signs, or hypoxemia.

**Conclusions:**

Lung ultrasound is a novel tool for the diagnosis of pediatric pneumonia with evidence supporting its validity and feasibility of implementation. Here we introduced a training program that resulted in a high level of inter‐sonographer agreement.

## 1 INTRODUCTION

Pneumonia remains a leading cause of mortality in children aged under 5 years worldwide,^1^ with most deaths occurring in lowresource settings.^2^ A barrier to receiving effective treatment is an early and accurate diagnosis. Current guidelines rely on clinical presentation and physical examination, with imaging deferred for use in treatment failure, severe, or ambiguous cases.^3,4^ While chest radiography is the current imaging standard for pneumonia, it is not obtained in all cases because it exposes children to unnecessary radiation. Moreover, the interpretation of chest Xrays suffers from high interobserver variability^4,5^ unless properly standardized.

Lung ultrasound (LUS) is now recognized as an alternative for imaging in pneumonia^6,7^ and does not suffer from the same radiation risk as do chest radiographs (CXR).^8,9^ Moreover, if the equipment and proper training are available, LUS can be easily obtained at the bedside. Our research group found that it was feasible to introduce LUS for the identification of consolidation in pediatric pneumonia to physicians with limited ultrasound experience in the low‐resource settings of Nepal^10^ and Peru.^11^ Indeed, a recent analysis from our group found that among a cohort of Peruvian children seeking care for an acute respiratory illness, LUS had the greatest ability to predict radiographically‐confirmed pneumonia when compared with clinical signs and symptoms, pulse oximetry and chest auscultation.^12^

A limitation to the use of LUS in epidemiological studies of pediatric pneumonia is a lack of consensus on what constitutes a primary endpoint pneumonia and an optimal scanning protocol^13-15^; unlike CXR, which has well‐established criteria for training and interpretation.^16^ Studies have shown that LUS may be able to replace CXR as an imaging modality for pneumonia^17^ with no change in clinical outcomes.^18^ As the use of LUS becomes more commonplace, we are left with the challenge of disseminating this skill for further research and clinical work. Here, we present our approach to training and standardization of general practitioners with limited ultrasound experience in Sylhet, Bangladesh.

## 2 METHODS

### 2.1 Study setting

The methodology for the 10‐valent pneumococcal conjugate vaccine (PCV10; Synflorix, GlaxoSmithKline) impact study in Sylhet, Bangladesh, was published previously.19 Briefly, the PCV10 impact study involved community‐ and facility‐based surveillance conducted in Sylhet, Bangladesh, between January 2014 and June 2018.19 The parent study sought to assess the impact of PCV10 against multiple endpoints including invasive pneumococcal disease, chest radiograph‐defined pneumonia, and LUS‐defined pneumonia, with multiple secondary aims including assessing the validity of LUS as a tool for pneumonia diagnosis in low‐resource settings.

### 2.2 Study design

Children aged 3 to 35 months presenting to the outpatient clinic of three subdistrict hospitals located in Beanibazar, Kanaighat, and Zakiganj, with cough or difficulty breathing, and diagnosed with clinical pneumonia were consecutively enrolled between June 2015 and September 2017.^19^ The first 2 months of the study (June‐July 2015) were dedicated to training and piloting protocols, and data collection began officially on August 2015. Informed consent for LUS was obtained at the inclusion of children into the study. IRB approval was obtained from the Institutional Review Board of the Johns Hopkins Bloomberg School of Public Health in Baltimore, MD; and the Ethical Review Committee of iccdr,b in Dhaka, Bangladesh. The full protocol for this study has been published elsewhere.^19^

### 2.3 Data collection

Following informed consent from the parent or guardian, child participants who met inclusion criteria underwent a standard clinical assessment. Child participants were assessed for clinical signs and symptoms and had lung auscultation, pulse oximetry, and imaging by LUS and CXR performed.19 Clinical exam was conducted by study physicians who followed a standardized clinical approach.19 Clinical pneumonia was defined as a history or presence of a cough or difficulty breathing, with one of the following: respiratory rate more than 50 breaths/minute for children aged 3 to 11 months and more than 40 breaths/minute for those aged 12 to 35 months, lower chest wall indrawing, persistent nasal flaring, head nodding or tracheal tugging, grunting, stridor, crackles or wheeze on chest auscultation, or wheezing audible without chest auscultation.19 All children meeting the definition of clinical pneumonia proceeded to have an anterior‐posterior CXR and underwent LUS. Diagnosis of primary endpoint pneumonia on CXR and LUS was established and standardized before study start (Table 1). Study physicians conducting LUS were blinded to the CXR results. Clinical diagnosis and LUS were conducted by different physicians, except at times of lowstaffing in the final 2 months of the study. Children were treated as part of the study, based on clinical signs and symptoms following World Health Organization (WHO) guidelines.19 All LUSs were performed using Sonosite Edge ultrasound machines (Sonosite/FujiFilm, Bothwell, WA) with an HFL38xi/13‐6 MHz linear transducer. A standardized approach was used to conduct LUS on all child participants. The hemithorax was divided into anterior, lateral, and posterior zones, and each zone was further divided into superior and inferior zones, for a total of 12 scanned zones.^20^ Each zone was scanned by sweeping the ultrasound probe both transversely and longitudinally.

**TABLE 1:** Lung ultrasound findings used in the Bangladesh Pneumococcal Conjugate Vaccine impact assessment

Quality	Interpretable	Ultrasound is interpretable for the presence or absence of endpoint consolidation, atelectasis, or interstitial abnormalities.
	Uninterpretable	Ultrasound quality is not interpretable for the presence or absence of endpoint consolidation, atelectasis, or interstitial abnormalities. Ultrasound does not have all 24 clips recorded.
Classification	Endpoint consolidation	Hypoechoic area or tissue pattern with loss or attenuation of distinct pleural lines.
	Air bronchogram	Fluid or inflammation along the bronchial walls. This is visualized on ultrasound as punctate hyperechoic or hypoechoic images.
	B-lines	Well‐defined hyperechoic comet‐tail artifacts arising from the pleural line, spreading down, indefinitely erasing A‐lines and moving with lung sliding when lung sliding is present.
	Pleural abnormality	Disruption along the pleural line that is not large enough to be measured as a consolidation.
	Shred sign	Disruption of the pleural line, caused by consolidation or pleural effusion, that forces the pleural line to become discontinuous and move below the level of the consolidation.
	Pleural effusion	Presence of fluid in the lateral pleural space between the lung and chest wall. This is visualized on ultrasound as hypoechoic images in the pleural space.
	Primary endpoint pneumonia	Presence of consolidation that measures ≥1 cm or greater than one intercostal space, or a pleural effusion with any of the following: consolidation <1 cm, ≥3 B‐lines, or presence of air bronchograms.
	Interstitial abnormalities	Presence of artifacts consistent with ≥3 B‐lines or pleural abnormalities.
	Atelectasis (small consolidations)	Presence of consolidation measuring <1 cm or <1 intercostal space.

Note: Description of findings on lung ultrasound and definition of endpoint pneumonia, interstitial abnormality, and atelectasis.

### 2.4 Definition of primary endpoint pneumonia on lung ultrasound

We defined primary endpoint pneumonia on LUS as either the presence of artifacts consistent with either a consolidation that measured ≥1 cm or was greater than one intercostal space, or a pleural effusion with any of the following: consolidation less than 1 cm, ≥3 B‐lines, or presence of air bronchograms.^21,22^ We did not differentiate between static and dynamic air bronchograms.

### 2.5 Training program

We used a training‐of‐trainers model to create a program to train and standardize study physicians on the use of LUS.^23^ Trainees were general practitioners from Bangladesh with limited experience in ultrasound and hired as study physicians in the PCV10 impact study. There were two types of experts for this study: remote and local experts. Remote experts were physicians from Johns Hopkins University in Baltimore, MD, who had extensive training and experience in LUS acquisition and interpretation. Local experts were study physicians who showed an enhanced aptitude for LUS and underwent additional training by remote experts on how to teach and supervise LUS. All LUS training was limited to the diagnosis of pediatric pneumonia.

Our training program consisted of two stages. In the first stage, remote experts conducted a 7 day, in‐person, didactic, and practical training session. Didactic training consisted of lectures on ultrasound physics and basics, use of knobs on ultrasound devices, applications of ultrasound, lung anatomy, and normal and abnormal findings on LUS. Practical training consisted of study physicians conducting ≥25 LUS on children under direct guidance by remote or local experts. Study physicians were then asked to review 25 LUS videos from images collected in Sylhet, Bangladesh, and passed if they had ≥80% accuracy in image interpretation when compared with remote experts. In the second stage, our remote or local experts directly supervised LUS scanning by the study physicians. This stage of training was completed when study physicians achieved ≥80% accuracy in image acquisition and interpretation when compared with remote or local experts. Upon successful completion of both stages of training, study physicians were considered standardized.

The first round of training took place on January 2015 in Sylhet, Bangladesh, with 17 study physicians participating. However, two additional training sessions were needed because of study physician turn‐over. The second round took place on November 2016 and we trained four new study physicians, with didactic training presented remotely by an expert in Baltimore and practical training conducted inperson by local experts in Sylhet. The third session took place on December 2016 and we trained an additional four new study physicians. Both didactic and practical training were conducted in‐person in Sylhet, Bangladesh by our remote experts. A refresher training session for all study physicians was conducted in March 2017.

Modifications to the training program were made based on feedback and data from the initial round of training. The first round of training required study physicians to have ≥80% accuracy in image interpretation when compared with the remote expert, while in subsequent rounds we required ≥85% accuracy.

### 2.6 Quality control

During the first 5 months of data collection, 100% of LUS scans were reviewed by remote expert sonographers using a cloud‐based image management system (Ultralinq Healthcare Solutions, New York, NY). Images were manually exported to Ultralinq by study physicians following a standardized process and back‐ups were stored locally in a hard drive in Sylhet, Bangladesh. Every month a quality control check was done to ensure all images were adequately uploaded to Ultralinq. At the end of this 5‐month period, all study physicians were considered standardized and allowed to conduct LUS independently, having achieved approximately 80% agreement with remote expert sonographers (Table 2). At this point, a two‐reader system was implemented, where one study physician would conduct and interpret the LUS and a second study physician would review the same LUS, within 24 hours, blinded to the first study physician’s interpretation. A remote expert reader, from a panel of three expert readers, would review discrepancies acting as an ombudsman to provide a final diagnosis. In addition, 20% of all LUS conducted each month were reviewed by remote expert sonographers as part of a quality control process to ensure standardization.

**TABLE 2:** Sensitivity and specificity, listed in columns two and three, of study physician interpretation of LUS, compared with expert readers as standard

Month	Number of scans	Sensitivity (%)	Specificity (%)	AUC (95% CI)	Percent agreement (%)
August 2015	30	60	92	0.76 (0.52-1.00)	87
September 2015	81	61	84	0.73 (0.63-0.83)	75
October 2015	222	50	91	0.70 (0.64-0.78)	81
November 2015	313	63	96	0.79 (0.74-0.85)	89

Note: Area under the ROC curve (AUC) is listed to represent the fit of diagnostic validity. Percent agreement is the agreement between the study physician and expert reader Abbreviations: AUC, area under the curve; CI, confidence interval; ROC, receiver operating characteristic curve.

### 2.7 Biostatistical methods

The primary analytical objective was to evaluate the agreement between sonographers in the assessment of LUS for the presence or absence of primary endpoint pneumonia. We used Cohen’s kappa coefficient (κ) to assess agreement between pairs of sonographers. A κ between 0.61 and 0.80 is usually considered a high agreement, and more than 0.8 is exceptional.^24^ A two‐way κ was calculated between two sonographers, and a three‐way κ was calculated among the two sonographers and a third, remote expert reader. We estimated 95% confidence intervals (CI) using standard methods for the two‐way κ and a bias‐corrected bootstrap method with 200 samples for the three‐way κ. We also calculated percent agreement between pairs of sonographers, and sensitivity and specificity to assess the sonographer’s ability to conduct and interpret LUS, using the expert reader as the gold standard. We conducted subgroup analyses by age, sex, presence of general danger signs, and categories of oxygen saturation (defined as a SpO_2_ ≤ 92%, 93%‐95%, and 96%‐100%). We used Stata version 15 (StataCorp, College Station, Texas) for statistical analyses.

## 3 RESULTS

Upon completion of the 2‐year study, 9051 pediatric LUS were conducted; 58% of child participants were males and 53% were less than 12 months of age. A total of 8053 pediatric LUS were retrospectively read by a second reader, and 1191 pediatric LUS were retrospectively read by a remote expert third reader during the study period. We present percent agreement, sensitivity, and specificity of LUS interpretation for the study sonographers compared with remote expert readers in Table 2. We found an 87% agreement during the first month of data collection, with high sensitivity and high specificity when compared with the remote expert reader. In the second month of data collection, the percent agreement decreased slightly, with low sensitivity and high specificity when compared with the remote expert reader. Over the next 2 months of the study, sensitivity and specificity improved to 63% and 96% respectively, with 89% agreement. After 5 months of remote experts reviewing 100% of LUS in November 2015, reaching an average total of 51 LUS studies each, study physicians maintained a percent agreement of 89% with remote expert readers. At this time, we moved forward with a two‐reader and quality control system as outlined above. In the second and third rounds of training, study physicians were able to achieve standardization in 2 months and it only required an average of 44 LUS studies each.

**FIGURE 1:**
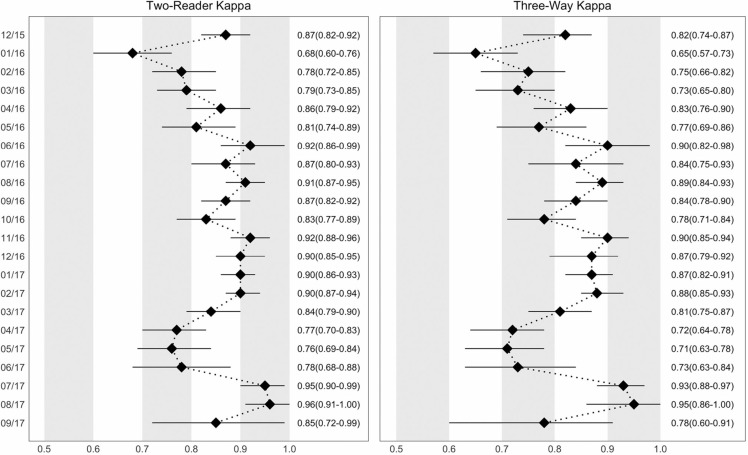
Agreement between sonographers by month over the course of the study; Sylhet, Bangladesh (2015‐2017). Left panel: two‐reader kappa, shows two‐way agreement between first and second readers. Right panel: three‐reader kappa with expert, shows three‐way agreement between first reader, second reader, and remote expert reader. The kappa agreement value is displayed on the x‐axis, and the month, from the beginning of the study to the end of data collection, is displayed on the y‐axis. The overall kappa for the specific month is plotted with a black diamond, with a line representing the 95% confidence interval. The kappa value and corresponding 95% confidence interval is displayed to the right of each figure

In Figure 1, we present the two‐way κ between study physicians and three‐way κ between study physicians and remote expert readers from December 2015 until study completed in September 2017. Upon initiation of the two‐reader system, there was an 95% agreement between the two study physicians, with a κ of 0.82 when both are compared with the remote expert reader. However, it dropped down to 89% with a κ of 0.69 in the next month and rose again to 97%, with a κ of 0.90 by June 2016 when compared with the remote expert, six months after initiation of independent LUS reading by study physicians. Percent agreement remained above 93% and the κ remained above 0.78 when compared with the remote expert for the next 9 months of the study. In April 2017, 16 months after initiation of independent LUS reading by study physicians, there was a lower agreement among study physicians and with remote expert sonographers (Figure 1). The agreement returned to pre‐April 2017 levels by July 2017 without intervention from study investigators and was maintained there on for the course of the study (Figure 1).

**FIGURE 2:**
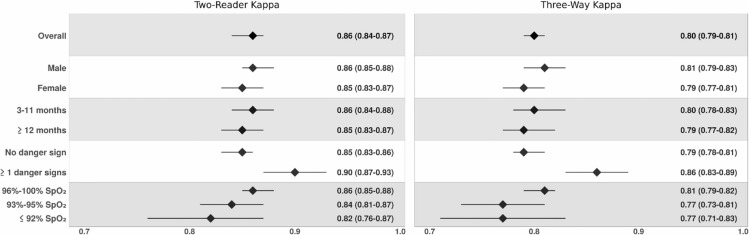
Overall agreement over the course of the two‐year PCV10 impact study; Sylhet, Bangladesh (2015‐2017). This figure lists the overall kappa, as well as kappa stratified by sex, child age, presence of one or more general danger signs (stridor, convulsions, inability to feed, decreased level of consciousness and waist‐to‐height ratio <−3 standard deviations), and oxygen saturation. The graph on the left, two‐reader kappa, shows two‐way agreement between first and second readers. The graph on the right, kappa expert, shows three‐way agreement between first reader, second reader, and a remote expert reader. The kappa agreement value is displayed on the x‐axis. The overall kappa for the stratification is plotted with a black diamond with a line representing the 95% confidence interval. The kappa value and corresponding 95% confidence intervals are displayed to the right of each figure

The overall κ, two‐way κ between study physicians and three‐way κ with remote expert readers, is present in Figure 2. Overall, a twoway κ of 0.86 (95% CI, 0.84‐0.87) was achieved between first and second readers. The κ was lower but still significant when a three‐way κ was conducted with a remote expert reader (κ = 0.80; 95% CI, 0.79‐0.81), and it remained high when stratified by age or sex of the child participants (Figure 2). In addition, when general danger signs were assessed, sonographers achieved a higher κ, when assessing children with at least one general danger sign (κ=0.90; 95% CI, 0.87‐0.93) compared with no danger signs (κ = 0.85; 95% CI, 0.83‐0.86) (Figure 2). While there were differences in κs among sonographers when stratified by SpO2, with κ improving as hypoxemia decreased, the agreement remained overall high (Figure 2).

## 4 DISCUSSION

We found that our training program was able to successfully train physicians in a low‐resource setting on the use of LUS to diagnose pediatric pneumonia. Our program achieved and maintained high two‐way kappa agreement between study physicians and three‐way kappa when both study physicians were compared with expert readers (0.86 and 0.80, respectively).

After completing a 1‐week standardized training program, followed by approximately 2 weeks of practical training, our study physicians achieved high sensitivity and specificity compared with expert sonographers. The low sensitivity, compared with an expert, in the first 4 months of the study improved as the study physicians improved their understanding of LUS and were able to practice independently. After 4 months of supervised scanning, study physicians began conducting LUS independently with the significant agreement between two study physicians or when compared with an expert. The agreement between study physicians and with the expert readers continued to improve throughout the course of the study, peaking after 6 months of independent scanning. There was a slight decrease in agreement for 3 months, after 16 months of independent LUS scanning by study physicians, likely attributed to a period of turnover with five experienced physicians leaving the group. The agreement remained high when analyzed as subgroups based on child age, sex and the presence of general danger signs or hypoxemia. These subgroups were chosen due to a possible increase in difficulty in scanning younger and more severely‐ill children. There was a difference in κ between the different SpO_2_, with κ improving as hypoxemia decreased, however, this difference was not significant and κ remained high in each individual stratum.

Our training program provides a potential solution to the need for standardized and validated training programs for LUS.15,25 According to the literature, current training programs have only assessed pre‐ and posttraining knowledge, not long‐term standardization. ^25^ WHO developed a methodology for CXR interpretation in the epidemiological study to allow for improved diagnosis of bacterial pediatric pneumonia and generalizability of epidemiological studies. ^16,26^ A similar consensus methodology for LUS training and standardization may allow for better application of LUS for the diagnosis of pediatric pneumonia in low‐resource settings.^15,27^

Our study has several strengths. First, to our knowledge, this is the largest study assessing the ability to train and standardize physicians on the use of LUS for the diagnosis of pediatric pneumonia.^9,28^ Second, we have collected clinical data on most of the child participants allowing subgroup analysis based on patient characteristics and clinical severity. Third, we were able to follow largely the same group of physicians throughout the 2‐year study, allowing us to see if there is a change in standardization as the study progressed.

Our study also has some potential shortcomings. First, our study was limited by the number of LUS completed in the first 6 months of data collection, potentially impacting our ability to assess the validity of our initial training program. However, over the course of the study, 8053 LUS were read by two physicians and 1191 read by a remote expert third reader during the study period, all showing high agreement. Second, there is no standard definition of primary endpoint pneumonia on LUS. We developed a definition based on current evidence, consistent with the WHO definition of pneumonia on CXR and our previous experience with LUS. Third, there is a lack of evidence around differentiating between viral and bacterial pneumonia using LUS. We attempted to make our definition more specific to bacterial pneumonia by introducing a size criterion to consolidations and including pleural effusions, but there is no current gold standard to differentiate between bacterial and viral pediatric pneumonia when using LUS. Fourth, our study required significant financial support to carry out training and standardization. While the costs of ultrasound machines are falling, the costs of training and standardization remain high and can limit the use in low‐resource settings.

## 5 CONCLUSION

LUS is a novel tool with emerging evidence supporting its potential to improve the diagnosis of pediatric pneumonia in resource‐poor settings. Data on the use of other imaging modalities used to diagnose pneumonia in children suggest that a standardized approach optimizes diagnostic accuracy. We have presented evidence on our program’s ability to achieve and maintain a high level of standardization among physicians with limited ultrasound knowledge. However, more research is needed on classification and diagnostic validity before LUS can be implemented more widely outside of a study setting.
